# Ecological niches and biogeography of nitrogen‐fixing plants in Europe

**DOI:** 10.1111/plb.70230

**Published:** 2026-05-14

**Authors:** N. Fahs, I. Axmanová, J.‐C. Svenning, T. Těšitelová, J. Padullés Cubino, I. Biurrun, J. A. Campos, J. Dengler, E. Garbolino, J. Těšitel

**Affiliations:** ^1^ Department of Botany and Zoology Masaryk University Brno Czech Republic; ^2^ Center for Ecological Dynamics in a Novel Biosphere (ECONOVO) Aarhus University Aarhus Denmark; ^3^ Botanical Institute of Barcelona (IBB‐CSIC) Barcelona Spain; ^4^ Department of Plant Biology and Ecology University of the Basque Country UPV/EHU Bilbao Spain; ^5^ Vegetation Ecology Research Group, Institute of Natural Resource Sciences (IUNR), ZHAW University of Applied Sciences Wädenswil Switzerland; ^6^ Plant Ecology, Bayreuth Center of Ecology and Ecological Research (BayCEER) University of Bayreuth Bayreuth Germany; ^7^ Higher Institute for Environmental Engineering and Management (ISIGE) Fontainebleau France

**Keywords:** Actinorhizal plant, biological nitrogen fixation, CHELSA Bioclim, ecological niches, EUNIS habitat classification, Europe, legumes

## Abstract

- Symbiotic nitrogen fixation by vascular plants represents a major pathway for nitrogen input in terrestrial ecosystems, fundamentally altering nutrient cycles and plant community dynamics. Nitrogen‐fixing plants comprise phylogenetically and physiologically distinct lineages whose ecological niches and responses to environmental gradients remain poorly resolved at continental scales. We investigated the geographic distribution and ecological responses of major nitrogen‐fixing lineages across Europe, focusing on legumes (inverted repeat lacking clade [IRLC], characterised by high symbiont regulation ability, and non‐IRLC) and actinorhizal genera.
- We analysed 707,673 vegetation plots (1970–2021) from the European Vegetation Archive to map lineage density at 30‐km resolution, assess habitat associations, model climatic drivers and evaluate distributions along environmental gradients using ecological indicator values.
- Non‐IRLC legumes predominated in Mediterranean scrublands and dry grasslands, whereas IRLC legumes extended into northern regions and mesic grasslands. Legumes were associated with high diurnal temperature range, high summer temperatures, low summer rainfall and low soil nitrogen and water availability—patterns pronounced in non‐IRLC legumes, but less distinct or even absent in IRLC legumes. Actinorhizal lineages showed disparate habitat associations and contrasting climatic responses, with temperature seasonality as the strongest predictor—positive for *Alnus* and Elaeagnaceae and negative for the other lineages.
- Our findings demonstrate fundamentally divergent ecological niches among European nitrogen‐fixing lineages, reflecting distinct evolutionary histories and physiological strategies. Enhanced symbiont regulation in IRLC legumes likely facilitates persistence where the benefits of nitrogen fixation are limited. Despite sharing a common adaptive trait, nitrogen‐fixing lineages have evolved different strategies to colonise various environments under diverse climatic conditions.

## Graphical abstract

Despite sharing N fixation as a common trait, European legume and actinorhizal plant lineages occupy strikingly divergent ecological niches shaped by their evolutionary histories and physiological adaptations. Advanced symbiont control allows IRLC legumes to expand into northern, mesic regions, while non‐IRLC legumes are more common in Mediterranean, dry climates.

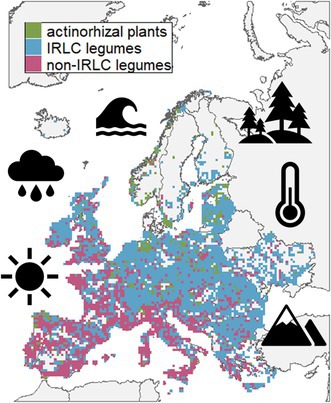

## INTRODUCTION

Plant growth is fundamentally constrained by the availability of essential nutrients, with nitrogen representing one of the most critical factors limiting plant productivity and ecosystem functioning in terrestrial ecosystems (Vitousek & Howarth 1991). While nitrogen comprises about 78% of the atmosphere, it remains largely inaccessible to plants until converted to bioavailable forms such as ammonium (NH_4_^+^) and nitrate (NO_3_^−^; Chanway *et al*. 2014). In terrestrial ecosystems, symbiotic biological nitrogen fixation by specialised prokaryotes represents the major pathway for nitrogen input, with nitrogen‐fixing bacteria inhabiting root nodules in vascular plants and exchanging fixed nitrogen for plant‐derived carbon compounds (Dilworth *et al*. 2008; Chanway *et al*. 2014). Nitrogen‐fixing plants serve as ecosystem engineers that fundamentally alter nutrient cycles (Rice *et al*. 2004; Lazzaro *et al*. 2018), influence plant community dynamics (Jacot *et al*. 2005) and drive primary succession across diverse global environments (Lawrence *et al*. 1967; Walker *et al*. 2003). They typically function as pioneers, enriching and stabilising nutrient‐poor soils (Boldt‐Burisch *et al*. 2015; Kucho *et al*. 2022).

Three principal groups of symbiotic nitrogen‐fixing bacteria exist: rhizobia, *Frankia* and cyanobacteria, each forming symbioses with distinct taxonomic groups of vascular plants (Tedersoo *et al*. 2018; Mathesius 2022). (i) Rhizobia are a paraphyletic group comprising more than 250 bacterial species across 18 genera. In Europe, they associate with Fabaceae (legumes). In both temperate and Mediterranean Europe, native legumes are predominantly herbs or dwarf shrubs; however, large shrub legumes are also notably common in the Mediterranean (Ardley & Sprent 2021; Loidi 2017, Večeřa *et al*. 2021). Legumes are capable of regulating nodulation efficiency, rewarding effective bacterial strains, restricting poorly performing ones and downregulating nodulation when external nitrogen is sufficient (Westhoek *et al*. 2021; Dagan *et al*. 2023). Within legumes, taxa belonging to the inverted repeat‐lacking clade (IRLC) can additionally control nodule numbers through autoregulation of nodulation (Mathesius 2022). They exhibit particularly high symbiont control by restricting nonspecific bacteria reproduction and promoting rhizobia‐specific polyploid bacteroids (Ardley & Sprent 2021; Mathesius 2022). These show increased transcription rates, leading to higher metabolic efficiency and more efficient N_2_ fixation. This lineage includes a broad diversity of European legumes, comprising many genera with temperate herbaceous representatives, such as *Trifolium*, *Vicia*, *Medicago* and *Lathyrus*, but also a few shrubs such as *Colutea arborescens* (Wojciechowski *et al*. 2004). (ii) Bacteria of the genus *Frankia* form symbioses with actinorhizal plants. In Europe, these plants represent a relatively small group of woody taxa. They evolved independently in related, yet distinct phylogenetic lineages within Fagales, Rosales and Cucurbitales and are mainly represented by the entire genus *Alnus*, as well as the species *Hippophae rhamnoides*, *Coriaria myrtifolia*, *Myrica gale* and *Elaeagnus angustifolia* (Tedersoo *et al*. 2018; Fahs *et al*. 2023). Compared to rhizobial plants, evidence of host regulation of actinorhizal symbiosis beyond the stage of initial infection remains limited and uncertain (Wall 2000; Froussart *et al*. 2016). (iii) Cyanobacterial taxa, such as *Nostoc* and *Anabaena azollae*, form specialised symbioses with Gunneraceae, gymnosperm cycads and the aquatic fern *Azolla*, respectively.

Large‐scale studies have identified climate and nitrogen availability as main determinants of spatial patterns of nitrogen‐fixing plants across different gradients (*e.g*. Pellegrini *et al*. 2016; Tamme *et al*. 2021; Acuña‐Acosta *et al*. 2024). Aridity and high temperatures are often associated with greater abundance and diversity of nitrogen fixers, relating to both optimal conditions for bacterial symbiont enzymatic functioning and nodulation, and the typically high water‐use efficiency of nitrogen‐fixing plants (Pellegrini *et al*. 2016; Liao *et al*. 2017; Acuña‐Acosta *et al*. 2024). By contrast, increasing soil nitrogen availability has frequently been shown to negatively affect the diversity and abundance of nitrogen fixers (Doby *et al*. 2022; Moreno‐García *et al*. 2024). However, several studies have indicated that overall distribution patterns can mask marked differences between rhizobial and actinorhizal lineages. Actinorhizal taxa tend to be more common in colder climates and at higher latitudes, whereas rhizobial plants predominate in warm temperate, tropical and arid regions (*e.g*. Menge *et al*. 2014; Ardley & Sprent 2021; Tamme *et al*. 2021). While such patterns are evident globally, local and regional studies have also shown high frequencies and ecological importance of legumes in cold environments (Herben et al. 2017; Jacot *et al*. 2005), and a recent global analysis further highlighted that their occurrence strongly depends on habitat type (Lepik *et al*. 2023). Taking into account various levels of symbiont regulation ability, growth form variability as well as strong differences in species‐specific ecological requirements, broad‐scale trends may be largely driven by dominant taxa, potentially obscuring substantial functional and ecological heterogeneity within nitrogen‐fixing lineages (evolutionarily close taxa, in this case mostly families). Lineage‐specific analyses of ecological niches may improve our understanding of how functional and environmental drivers shape their biogeographic distribution.

We used vegetation plot data from an extensive European vegetation plot database (European Vegetation Archive, Chytrý *et al*. 2016) to analyse the ecological niches of nitrogen‐fixing plants in Europe, with explicit consideration of lineage‐specific differences. Unlike incidence‐based datasets, vegetation plot data provide information on species co‐occurrence and their relative importance in plant communities (Bruelheide *et al*. 2019), further allowing the integration with ecological indicator values (Dengler *et al*. 2023) that provide community‐scale ecological characteristics. We aimed to compare the biogeography of nitrogen‐fixing lineages in terms of their (i) relative importance within their distribution range, (ii) habitat associations and (iii) prevalence along environmental gradients. We expected to observe differentiations in distribution patterns, habitats and positions on principal environmental gradients not only between legumes and actinorhizal plants but also within them. Specifically, we hypothesised that IRLC legumes will display broader ecological niches, also including habitats with higher nutrient availability compared to non‐IRLC legumes due to their advanced regulation of the rhizobial symbiosis. In actinorhizal plants, we expected diverse, lineage‐specific patterns in relation to environmental gradients.

## MATERIALS AND METHODS

### Vegetation data

Our study is based on a dataset of 1,122,281 vegetation‐plot records from the European Vegetation Archive (EVA; Chytrý *et al*. 2016; project number: 137; date of download: 26‐11‐2021). Details on data stratification and filtering are provided in Appendix S1. All plots were classified into EUNIS (European Nature Information System) habitat types at the highest hierarchical level using the EUNIS‐ESy expert system (Chytrý *et al*. 2020). This level contains the categories coastal saltmarshes (M), coastal sand and cliff habitats (N), wetlands (Q), grasslands (R), heathlands, scrub and tundra (S), forests (T), inland sparsely vegetated habitats (U) and anthropogenic habitats (V). Where the data permitted, plots were further assigned to intermediate habitat types (*e.g*. ‘dry grasslands’) and, when possible, to the most detailed level (*e.g*. ‘perennial rocky grassland of central and south‐eastern Europe’). Strongly synanthropic habitats (entire class V), non‐native forest plantations and transitional habitats (*e.g*. forest clearings, recently felled areas) were excluded (see Table S2.5 for details), as these are subject to recent intensive human disturbance, and our study focuses on natural and semi‐natural vegetation. The final dataset contained 707,673 vegetation plots, of which only those classified at least at the intermediate level (486,808) were used for the habitat analysis.

### Classification of symbiotic lineages

We defined symbiotic lineages corresponding to the main plant families that include nitrogen‐fixing taxa, while recognising that not necessarily all genera within these families form symbioses. Among actinorhizal taxa, we analysed the genus *Alnus* and nitrogen fixers within the families Myricaeae, Elaeagnaceae and Coriariaceae. *Alnus glutinosa* was analysed separately from the other *Alnus* species to account for its disproportionately high abundance in the dataset (see Results). In this context, *A. glutinosa* includes the recently described species *A*. *lusitanica*, and *A. incana* includes *A. rohlenae* (Vít *et al*. 2017). Due to their very low frequency in the dataset, we included nitrogen‐fixing Rhamnaceae only in the overview of species occurrences in habitat types (see Appendices S2.1–S2.3). We subdivided Fabaceae into IRLC and non‐IRLC lineages to account for differences in the regulation of nitrogen fixation symbiosis. For the assessment of their occurrence in habitat types and along ecological gradients, we also analysed legumes and actinorhizal plants collectively. Cyanobacteria‐associated nitrogen fixers were excluded from the present study due to their infrequency in European vegetation.

### Species nomenclature

We excluded all non‐vascular species and records not identified to at least the family level. Species names and ranks were standardised using the Euro+Med PlantBase checklist (Euro+Med 2023). Intraspecific taxa were merged to the species level, and species were grouped into aggregates following the EUNIS‐ESy expert system classification (Chytrý *et al*. 2020). Due to inconsistent separation of vegetation layers in the data, we did not differentiate them in our analysis. Instead, we aggregated individual species cover values within plots across layers, accounting for potential overlap using the Jennings–Fischer formula (Jennings *et al*. 2009; Fischer 2015).

The nitrogen‐fixation classification followed Fahs *et al*. (2023), available in the FloraVeg.EU database (https://floraveg.eu/; see Chytrý *et al*. 2024), with taxa designated as having symbiosis with rhizobia (legumes), symbiosis with *Frankia* (actinorhizal plants) or not being nitrogen‐fixing. Species with symbiosis marked as ‘likely’ and ‘unlikely’ in Fahs *et al*. (2023) were classified as non‐nitrogen‐fixing, as their status is not certain. Due to uncertainty, we also excluded all Zygophyllaceae from the analysis. Taxa were retained at the finest available resolution (usually species); genus‐ or family‐level records were kept if the nitrogen‐fixation status within Europe was unambiguous. This was the case at the family level for Fabaceae, Myricaceae, Coriariaceae and Elaeagnaceae and at the genus level for *Alnus* and *Colletia*. Information on genera belonging to the IRLC legumes was obtained from Wojciechowski *et al*. (2004), and the assignment of species to this category can be found in Table S2.1.

### Environmental variables

We obtained climatic data from CHELSA Bioclim (bio1‐bio19; Karger *et al*. 2017, 2018) at 30‐arc‐second resolution for each plot. We reduced the number of variables representing temperature and precipitation patterns using RDA forward selection to mean diurnal air temperature range (bio2), temperature seasonality (bio4), mean daily mean air temperatures of the warmest quarter (bio10), annual precipitation amount (bio12), precipitation seasonality (bio15) and mean monthly precipitation amount of the warmest quarter (bio18). The variables entering the RDA analysis were centred and scaled to unit variance. This selection accounted for 95.3% of the variation in the climate dataset. Precipitation variables were log‐transformed for the modelling analysis. For more details on the selection and pairwise correlation of variables, see Appendix S3.

Atmospheric nitrogen data were acquired from the EMEP database (https://emep.int/mscw/mscw_moddata.html) at 0.1° resolution and summed annually (NO_x_ and NH_3_). For pre‐1990 data, we applied correction factors to 1990 nitrogen data following the methods by Duprè *et al*. (2010): 1980–89 (1.1) and 1970–79 (1.3). Each record was assigned the mean and total atmospheric nitrogen value from the 10 years preceding its sampling date. Since nitrogen‐fixing plant occurrence did not show any relevant pattern in relation to atmospheric nitrogen data, we excluded these variables from our final analyses (for model results including atmospheric nitrogen, see Appendix S6.4).

### Ecological indicator values

To analyse fine‐scale environmental responses that may not be captured by macro‐environmental variables, we used empirically derived indicator values (Ellenberg 1974), namely, Ecological Indicator Values for Europe (EIVE) for soil nitrogen, soil moisture, soil reaction, temperature and light (Dengler *et al*. 2023, values scaled 0–10). For species aggregates lacking EIVE data, we calculated mean values using all constituent species (according to Chytrý *et al*. 2020) with available data. In the final dataset, 69% of taxa contained EIVE information for at least one category. However, because taxa without EIVE information were typically rare ones, EIVE data were available for 98% of all taxa × plot observations. We computed unweighted mean EIVE values for each plot separately for symbiotic lineages as well as for legumes and actinorhizal plants and all nitrogen fixers together. To avoid circularity, the mean EIVE of a plot for any given focal group was calculated using only the values of species that did not belong to that specific group.

### Statistical analysis

#### Spatial patterns and habitat associations

We determined the total cover per plot by summing the absolute covers of all individual species per plot, not considering their overlap. We then calculated the relative cover of each symbiotic lineage per plot by summing the relative cover values of all species within that lineage (each species' cover divided by the total cover for the plot). Then, we established a 30‐km grid across the study area in Europe and calculated the mean relative cover for each symbiotic lineage over all plots per grid cell. Mapped colour classes were defined using equal quantiles for each group and calculated after excluding cells with 0% mean relative cover. This way we created lineage‐specific occurrence maps across all habitats (Fig. 2) and also created habitat‐specific maps based on broad EUNIS habitat types for all symbiotic lineages (see Figs S4.2–S4.8) and maps representing the relative share of the species pool per grid cell for legumes and actinorhizal plants (see Fig. S4.1), and, finally, maps representing mean values of the climatic predictors (see Figs S5.1–S5.6).

#### Environmental drivers

We analysed the effects of climate and atmospheric nitrogen on the prevalence of a symbiotic lineage using boosted regression trees (BRTs). BRTs are particularly suited for large‐scale vegetation data as they combine regression trees with boosting algorithms to capture complex, non‐linear relationships (De'ath 2007; Viana *et al*. 2022). Our initial models included both climatic variables and atmospheric nitrogen (Fig. S6.5 and Table S6.2). However, as atmospheric nitrogen showed no relevant trends, it was excluded from the final analysis. The final BRT models focused exclusively on the six climatic variables described above (bio2, bio4, bio10, bio12, bio15, bio18; precipitation values log‐transformed as described in Table S2.4). Models were fitted using the *gbm.step* routine (Elith *et al*. 2008) in the ‘dismo’ package (Hijmans *et al*. 2011) with R version 4.3.0 (R Core Team 2023). We set the Bernoulli distribution as the error distribution. To account for the quantitative nature of the relative cover values, we implemented group‐specific plot weighting following Yu *et al*. (2020): plots without species of the focal group received a weight of 1, while others were weighted by the group's relative cover. Plots with species of the focal group were scaled as:

$$\text{Scaled weight}=\frac{\text{relative cover}\;-\;\min.\;\text{relative cover within the group}}{\max.\;\text{relative cover within the group}\;-\;\min.\;\text{relative cover within the group}}*100+1$$

The mean of median presence weights across the BRTs was 5.75. Thus, group presences had relatively higher weights in the BRT models compared to absences. We set the training/test subset ratio to 0.5, used a learning rate of 0.001 and applied a tree complexity of 5. The optimal number of regression trees was determined through automated 10‐fold cross‐validation, with folds selected via random stratified sampling (to ensure a consistent proportion of presences and absences across all folds) as implemented in the gbm.step routine (Elith *et al*. 2008). This cross‐validation procedure was used to identify the model size that minimised predictive deviance. For further details on the methods, see Supplementary Material S6. Model evaluation statistics are shown in Table S6.1. From the final BRTs, we extracted the relative importance values of individual predictors and created partial dependence plots to visualise predictor–response relationships (see Figs S6.1–S6.3). To check for spatial autocorrelation in model residuals, we calculated Moran's *I* statistics across distance classes (defined by Sturges's rule) using the *‘corrlog’* function from the *pgirmess* package (Giraudoux 2005; for details on the methods, see S6).

To evaluate the distributions of lineages along environmental gradients we tested whether EIVE values differed significantly between plots with and without specific symbiotic lineages, we employed a modified permutation test following Zelený & Schaffers (2012). At each iteration, species‐level EIVE values were randomly reassigned, from which plot‐level mean EIVE values were recalculated. We then derived the mean difference in EIVE between plots with and without species of a given symbiotic lineage (Diff_rand_). This process was repeated 999 times to construct a null distribution of expected differences (see Appendix S7). The observed difference in mean EIVE (Diff_real_) was then compared against this distribution. We quantified the standardised effect size (SES) as follows: SES = (Diff_real_ − mean(Diff_rand_))/sd(Diff_rand_). Positive SES values indicate that plots containing the symbiotic lineage had higher EIVE than plots without it, whereas negative SES values indicate the opposite. The associated p‐value was obtained by calculating the proportion of randomisations in which Diff_rand_ exceeded Diff_real_ in the direction indicated by SES.

## RESULTS

### Species numbers and geographic patterns

**Fig. 1:**
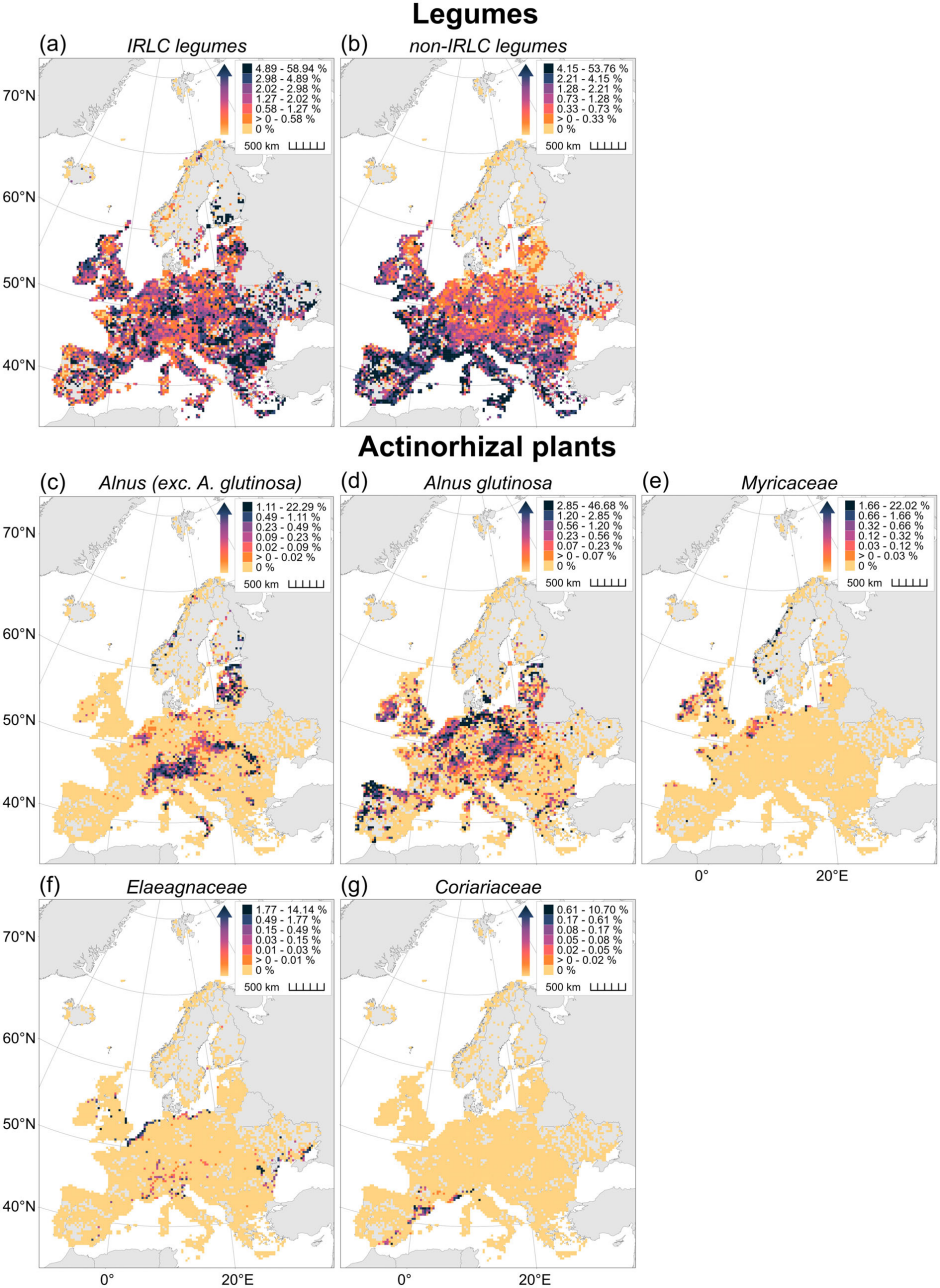
Mean relative cover of nitrogen‐fixing lineages (a) IRLC legumes, (b) non‐IRLC legumes, (c) *Alnus* spp. (except *Alnus glutinosa*), (d) *A*. *glutinosa*, (e) Myricaceae, (f) Elaeagnaceae and (g) Coriariaceae across Europe. Data are presented as mean values per 30 km grid cell (Lambert Azimuthal Equal Area projection, EPSG:3035). Only grid cells with ≥3 vegetation plots were included in the analysis. Colour class scales are based on equal quantiles and differ between plots.

Our dataset included 706 legume species (424 IRLC and 282 non‐IRLC), along with 15 actinorhizal plant species (6 *Alnus* species, 5 species from the Elaeagnaceae family, 2 from Myricaceae, 1 from Rhamnaceae, 1 from Coriariaceae). The most frequently recorded legumes were *Trifolium repens* (in 13.4% of all records and 1.2 times more frequent than the next most frequent species), *T. pratense*, *Lotus corniculatus*, *Lathyrus pratensis* and *Vicia cracca*; all these species, except *L. corniculatus*, belong to the IRLC clade. Among actinorhizal plants, *Alnus glutinosa* dominated the records (in 3.7% of all records and 3.7 times more frequent than the next species), followed by *A*. *incana*, *Hippophae rhamnoides*, *Myrica gale* and *A*. *viridis* (for the full species lists with their occurrence in the most common habitats, see Table S2.1). Nitrogen‐fixing plants occurred all across Europe but showed distinct biogeographic patterns between lineages (Fig. 1). Legumes consistently had a higher relative cover than actinorhizal plant lineages. Non‐IRLC legumes reached the highest relative cover in Mediterranean, Western Atlantic and Eastern Continental and Steppic regions, while IRLC legumes showed no clear spatial pattern. Actinorhizal plants, despite lower overall cover, were also distributed widely from southern Iberia to northern Norway, with *Alnus glutinosa* (including *A*. *lusitanica*) exhibiting the broadest distribution. Other *Alnus* species peaked in the Alps and Carpathians, extending northwards to the Baltic States and Fennoscandia. Myricaceae concentrated in Atlantic regions, Elaeagnaceae along the Northern Atlantic, Baltic coasts, Alps and the Black Sea, while Coriariaceae remained largely restricted to western Southern Europe, mostly confined to the Mediterranean Sea.

### Habitat associations

**Fig. 2:**
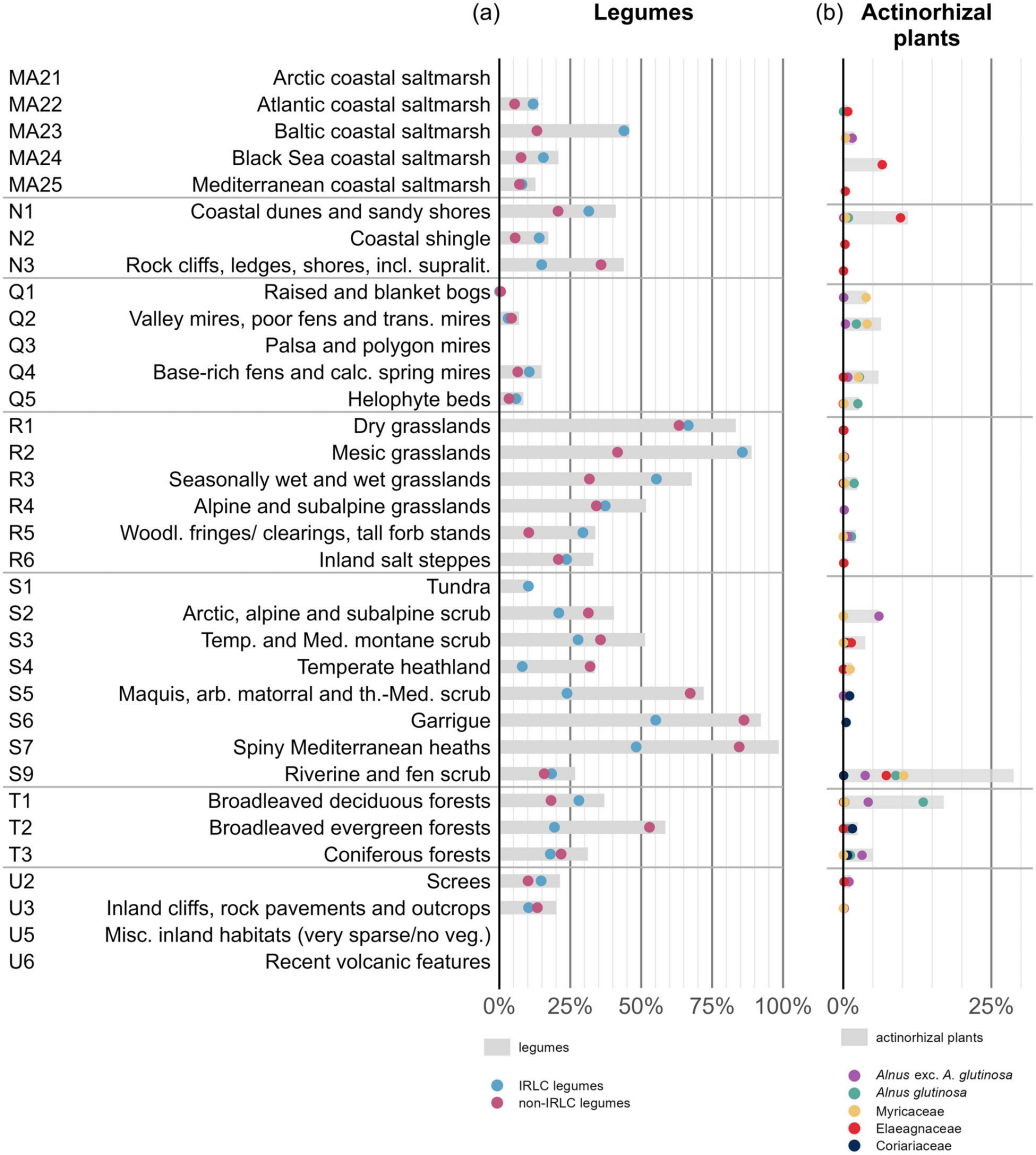
Proportion of vegetation plots containing at least one occurrence of (a) a legume and (b) an actinorhizal plant across European (EUNIS) habitat types (intermediate level). Bars show the overall proportion of plots containing a legume or actinorhizal plant, respectively. Dots indicate the proportion of plots containing at least one record of a plant of an individual symbiotic lineage within. Note the different ranges of the x‐axes in (a, b). For detailed information of species occurrence in habitat types, see Appendix S2.1.

Habitat associations differed between legumes and actinorhizal plants and symbiotic lineages within (Fig. 2). Legumes were frequent across most habitats except wetlands, with the highest frequencies in Mediterranean scrubs (almost 100% of plots of spiny Mediterranean heaths) and grasslands (50%–80% of plots). IRLC legumes dominated in mesic and wet grasslands, non‐IRLC legumes dominated in scrub habitats except tundra and riverine and fen scrubs, particularly in Mediterranean scrubs. Actinorhizal plants were most abundant in riverine and fen scrubs (>25% plots, driven by multiple lineages) and broadleaved deciduous forests (>15% plots, mainly *Alnus glutinosa* and other *Alnus* species). *Alnus* species (except *A. glutinosa*) were also common in arctic, alpine and subalpine scrub and in screes. *A*. *glutinosa* grew in most wetland types (excluding palsa and polygon mires). Myricaceae occurred in wetlands with even higher frequencies. Elaeagnaceae colonised coastal dunes and sandy shores, Black Sea coastal salt marshes and temperate and Mediterranean montane scrubs. Coriariaceae were most abundant in broad‐leaved evergreen forests and Mediterranean scrub. For an overview of the most common nitrogen‐fixing species per EUNIS habitat types (level 1 and 3), see Tables S2.2 and S2.3.

### Occurrence along environmental gradients

Climatic modelling showed differing dependencies among nitrogen‐fixing lineages (Table 1 and Fig. S6.1). Both IRLC and non‐IRLC legumes were positively related to mean diurnal temperature range and mean summer temperature. This pattern was more pronounced in IRLC legumes. Temperature seasonality was identified as the strongest predictor for non‐IRLC legumes, which, however, showed a bimodal response (Table 1 and Fig. S6.1b). This predictor had a negative effect on IRLC legumes, but its relative influence was rather weak. In addition, both legume lineages were associated with low summer precipitation. Across actinorhizal lineages, temperature seasonality was the best predictor, showing a positive association for *Alnus* spp. (except *A. glutinosa*) and Elaeagnaceae and a negative association for *A. glutinosa*, Myricaceae and Coriariaceae (Table 1; Figs S6.2 and S6.3). All actinorhizal lineages, except Myricaceae, showed a positive association with mean summer temperature. Other important predictors for actinorhizal lineages included mean diurnal temperature range and summer precipitation, which also showed inverse relationships among groups. Spatial correlograms of model residuals showed little to no residual spatial autocorrelation across most nitrogen‐fixing lineages (Fig. S6.5), with mean Moran's *I* values close to zero and rapidly converging towards spatial independence. Weak, short‐range autocorrelation was observed for a few lineages (Myricaceae, Elaeagnaceae and Coriariaceae), but effect sizes were modest and did not persist across broader spatial scales, suggesting that remaining spatial structure is unlikely to bias model inference. However, the predictive power for these geographically restricted lineages is inherently constrained by their limited ranges, where climatic predictors are more likely to be confounded with spatial location.

**Table 1:** Relative influence (%) of climatic predictors on the abundance‐weighted occurrence probability of nitrogen‐fixing symbiotic lineages in Europe, derived from BRTs.

	Legumes		Actinorhizal plants				
	IRLC	n‐IRLC	Aln	*A. glu*	Myr	Ela	Cor
Mean diurnal air temperature range (°C)	20.6	38.5	12.3	18.2	14	16.7	13.5
Temperature seasonality (°C/100)	30	11.9	26	20	27.1	30.9	18.3
Mean daily mean air temperatures of the warmest quarter (°C)	17.9	21.3	18.1	22.5	14.2	7.8	14.7
Annual precipitation amount (log(mm))	3	9	15.5	15.7	21.5	15.7	16.6
Precipitation seasonality (log(mm))	14	5.3	8.4	9.7	10.6	17.9	22.2
Mean monthly precipitation amount of the warmest quarter (log(mm))	14.5	14.1	19.8	13.9	12.7	11	14.6

The score values of each model are scaled, summing up to 100% (numbers displayed are rounded). Red colours indicate a positive relationship, blue colours a negative relationship and yellow colours a complex relationship, based on the partial dependence plots, presented in Appendices S6.1–S6.3.

*A. glu*, *Alnus glutinosa*; Aln, *Alnus* spp. (except *A. glutinosa*); Cor, Coriariaceae; Ela, Elaeagnaceae; IRLC, IRLC legumes; Myr, Myricaceae; n‐IRLC, non‐IRLC legumes.

**Fig. 3:**
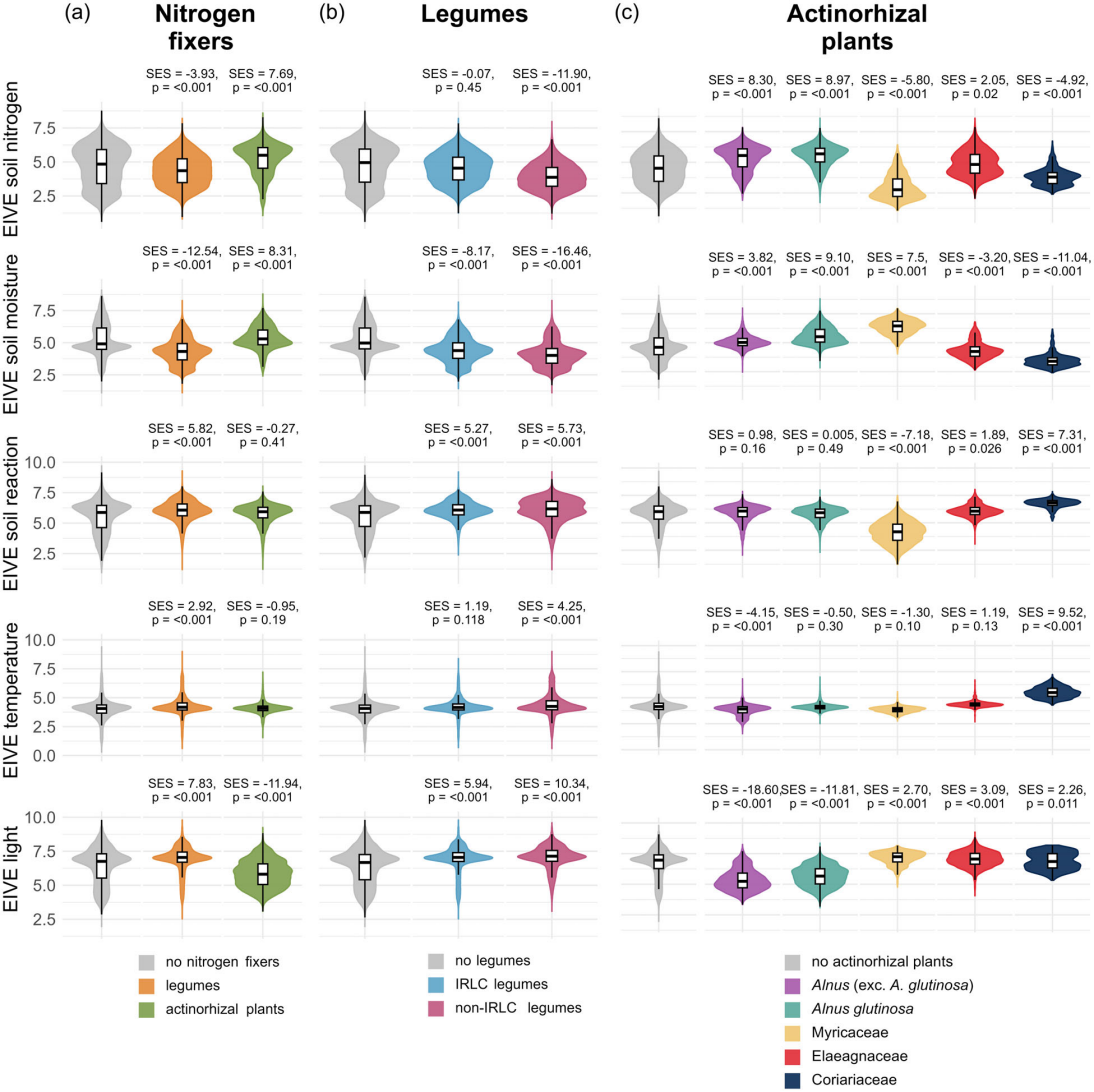
Violin plots depicting the distribution of Ecological Indicator Values for Europe (EIVE) across vegetation plots with (a) no nitrogen fixers, legumes and actinorhizal plants; (b) no legumes, IRLC legumes and non‐IRLC legumes; and (c) with no actinorhizal plants, *Alnus* spp. (except *A. glutinosa*), *A*. *glutinosa*, Myricaceae, Elaeagnaceae and Coriariaceae across Europe. Boxplots indicate medians, quartiles and non‐outlier ranges. SES values represent the standardised effect size of differences between EIVE means across plots with and without nitrogen‐fixing lineages, with corresponding *P*‐values indicating statistical significance. For Mean EIVE values for all lineages and null‐model histograms, see Table S7.1 and Fig. S7.1–S7.9, respectively.

Comparisons of mean ecological indicator values between plots with and without nitrogen‐fixers revealed significant differences (Fig. 3). Legumes overall occurred under slightly lower nutrient conditions than expected by chance (Fig. 3a), driven by non‐IRLC legumes (Fig. 3b; mean EIVE soil nitrogen value: 3.9), whereas IRLC legumes showed no significant difference (mean EIVE soil nitrogen value: 4.5). Both legume lineages were associated with drier conditions, elevated soil pH and light availability. Non‐IRLC legumes were also associated with significantly higher temperature values. Actinorhizal lineages showed more variable patterns: most were associated with higher soil nutrients, moisture and light availability than expected by chance, but specific lineages deviated from this trend (Fig. 3c). Myricaceae and Coriariaceae occurred under lower nutrient conditions than expected by chance (mean EIVE soil nitrogen values: 3.0 and 3.8, respectively), Elaeagnaceae and Coriariaceae under lower moisture conditions (mean EIVE soil moisture values: 4.3 and 3.5, respectively) and *Alnus* species under lower light conditions (mean EIVE light values: 5.4 for *Alnus* spp. except *A. glutinosa* and 5.6 for *A. glutinosa*). Significant associations with soil pH were observed only for Myricaceae (negative) and Coriariaceae (positive), whereas only *Alnus* spp. (except *A. glutinosa*; negative) and Coriariaceae (positive) showed significant deviations from temperature patterns expected by chance.

## DISCUSSION

Our study provides a detailed survey of the biogeography and ecological preferences of nitrogen‐fixing plants in Europe. Our functional and family‐level analyses demonstrate previously unquantified lineage‐specific patterns, reflecting distinct evolutionary and physiological adaptations. Clear contrasts emerged not only between legumes and actinorhizal plants but also among symbiotic lineages within them. Climate, light availability and soil conditions were reaffirmed as key drivers of their distributions, yet the specific responses varied strongly between nitrogen‐fixing plant lineages.

### Legume distribution, habitat and ecological preferences

Across Europe, legumes were most frequent in open habitats such as grasslands and Mediterranean shrublands (Fig. 2a), consistent with their strong association with high light availability (Fig. 3a; Taylor & Menge 2018) required to sustain carbon‐rich photosynthate exchange with symbiotic bacteria (Minchin & Witty 2005; Ardley & Sprent 2021; Tamme *et al*. 2021). While legumes also occur in European forests (Fig. 2a), they generally grow in light forest margins and open stands. This is particularly evident in the Mediterranean (*e.g. Cytisus hirsutus, Hippocrepis emerus, Calicotome villosa*) but also open temperate forests (*e.g. Genista tinctoria, Vicia sepium*, *Trifolium lupinaster*; see Table S2.2). A notable exception is *Lathyrus vernus*, the only legume species identified as a true forest specialist (Heinken *et al*. 2022), able to grow even in closed calcareous beech forests (Table S2.2). This general dependence on light, and the resulting restriction of legumes to the more open‐structured Mediterranean forests, leads to a much more pronounced north–south gradient of legume occurrence in forests (Fig. S4.7a,b), compared to grasslands (Fig. S4.5a,b) and across habitats (Fig. 1a,b). Interestingly, native European legume taxa generally do not reach tree height, and shrubby legumes are predominantly confined to Mediterranean climates. In contrast, several introduced tree species have become invasive across large areas of Europe, including *Acacia* spp. and *Robinia pseudacacia*, which have proven particularly successful in transforming native ecosystems (Souza‐Alonso *et al*. 2017; Lazzaro *et al*. 2018).

Legumes in Europe, in general, were negatively associated with soil moisture and wet habitats (Fig. 2a and Fig. 3a). This supports the well‐established hypothesis that they are adapted to—at least seasonally—drought‐prone environments (*e.g*. Liao *et al*. 2017; Gei *et al*. 2018; Tamme *et al*. 2021). Legumes often exhibit morphological and physiological adaptations that enhance drought tolerance. Many species possess photosynthetic stems, which are particularly common in the family (Nilsen 1995), providing additional carbon gain that helps mitigate the effects of drought while allowing continued photosynthesis even after leaf shedding, thereby reducing transpiration (Ávila‐Lovera *et al*. 2017). Furthermore, their typically pinnate or bipinnate leaves offer flexibility under water stress, as individual leaflets can be shed, when necessary, to conserve water without entirely halting photosynthetic activity (Givnish 1978). In dry conditions, rhizobial nodulation enhances water‐use efficiency through osmotic adjustment and elevated photosynthetic efficiency of leaves supplemented by sufficient nitrogen (Inostroza *et al*. 2015; Adams *et al*. 2016; Schwab *et al*. 2023). Another strategy common, particularly among Mediterranean legumes, is to escape drought through a therophytic life cycle, completing growth and reproduction within short, favourable periods (Rochon *et al*. 2004). Such a life cycle may also be facilitated by photosynthetic efficiency, allowing fast growth. Particularly in grazed systems, drought and disturbance create microsites that further facilitate legume establishment (Merou *et al*. 2013).

We found notable differences between IRLC and non‐IRLC legumes in their spatial distributions, habitat associations and responses to environmental gradients. The general legume association with high summer temperatures and dry conditions is less pronounced in IRLC legumes (Table 1; Fig. 3; Fig. S6.1), which is reflected by more variable latitudinal patterns than in non‐IRLC legumes (Fig. 1). The capacity of IRLC legumes to maintain tightly regulated, highly efficient symbioses likely offsets the costs of nodulation even under suboptimal conditions. This is in contrast to non‐IRLC species, for which reduced nitrogenase activity in cold environments may diminish symbiosis benefits (Körner 2016). IRLC legumes are widely distributed in temperate climates worldwide (Wojciechowski *et al*. 2004). Species of *Trifolium*, *Lathyrus* and *Vicia* extend into northern Europe and even arctic habitats, while *Oxytropis* comprises many perennial legumes specialised to cold environments (see Table S2.1). However, a few non‐IRLC legumes are also able to grow in cold climates, including *Lotus* species and various genera of the tribe Genisteae (*e.g. Cytisus*, *Adenocarpus* and *Genista*) abundant in Mediterranean mountains (Loidi 2017). IRLC legumes also showed no preference for specifically nitrogen‐poor conditions (Fig. 3b), which are typically viewed as favourable for rhizobial plants. Species such as *Trifolium pratense*, *Trifolium repens*, *Vicia cracca* and *Lathyrus pratensis* are characteristic components of lowland hay meadows and other mesic habitats. Mesic grasslands host IRLC legumes in particularly high densities (Fig. 2a), most likely reflecting particular advantages of their regulatory precision of the rhizobial symbiosis in disturbed systems with spatially and temporally fluctuating nutrient availability and competitive pressure (Klaus *et al*. 2016).

### Actinorhizal plant distribution, habitat and ecological preferences

Our findings reveal no uniform response among actinorhizal plants to environmental predictors, highlighting their ecological and phylogenetic diversity. Contrary to global patterns (Tamme *et al*. 2021), we did not find universal negative associations between actinorhizal plant occurrence and precipitation or temperature across Europe, but lineages displayed distinct responses to these factors. Interestingly, temperature seasonality emerged as a key predictor across all lineages which did, however, differ in the direction of their response (Table 1). Most actinorhizal plants were concentrated in coastal regions with balanced oceanic climates, whereas *Alnus* spp. and Elaeagnaceae also extended into alpine and continental zones characterised by high temperature seasonality (Fig. 1d–h). While not previously recognised as an important predictor of European actinorhizal plant distribution, temperature seasonality has recently been suggested as a major driver for the distribution of *Alnus* species in China (Yang *et al*. 2025).

Globally, actinorhizal plants are recognised as early‐successional pioneers in disturbed, nitrogen‐limited sites, enriching soils until their replacement during succession (Chapin *et al*. 1994; Walker *et al*. 2003; Ardley & Sprent 2021). This role is less pronounced in Europe, with some actinorhizal plants indeed occupying successionally young habitats linked to disturbance (*e.g. Alnus glutinosa* in riparian and swamp forests; *Hippophae rhamnoides* in coastal dune and riparian scrub; Fig. 2); however, these are not always strictly nitrogen‐poor. In addition to the anticipated occurrence of *A. glutinosa* in nutrient‐rich sites, other *Alnus* species and Elaeagnaceae are also common in such environments (Fig. 3c). Actinorhizal plants are typically regarded as obligate nitrogen fixers with limited regulatory control over their symbionts, potentially continuing fixation under unfavourable conditions (Andrews *et al*. 2011; Menge *et al*. 2014). Under high nitrogen availability, fixation becomes superfluous, imposing unnecessary carbon costs on the plant to support the symbiont. Similarly, under low phosphorus conditions or drought stress, the plant must divide limited resources between its own physiological requirements and those of the symbiont. Despite these constraints, occurrences across wide gradients of nutrients and moisture (Fig. 3c) suggest that the weakly regulated symbiotic fixation may remain profitable under highly variable resource conditions. However, the energetic costs of supporting symbionts may constrain competitive ability, in particular sensitivity to competition for light (Sprent & Scott 1979). As a result, actinorhizal plants tend to inhabit extreme environments characterised by cold temperatures (alpine scrubs), high moisture levels (riverine and fen scrubs), acidity (bogs and mires), low nutrient levels (dunes, sandy shores and mires) or salinity (saltmarshes), where competition for light is limited (Fig. 2). Certain species show specific adaptations to these habitats, such as negatively gravitropic roots in *Myrica gale*, facilitating oxygen access above waterlogged soils, enabling growth in acidic, waterlogged wetlands (Skene *et al*. 2000).

*Alnus* spp. and *Coriariaceae* also frequently occur in forests with closed canopies; nevertheless, their sensitivity to competition may affect their population dynamics. For example, *A. glutinosa* regeneration requires high light availability (and suitable hydrological conditions), with seedlings failing to establish beneath the canopy of larger overstorey trees (Natlandsmyr & Hjelle 2016). Thus, *A. glutinosa* typically regenerates from open gaps formed by disturbance or dieback of old *Alnus* trees (Natlandsmyr & Hjelle 2016; Douda *et al*. 2020) or from coppice shoots in managed forests (Claessens *et al*. 2010).

### Future dynamics

Ongoing global change is likely to reshape the distribution and ecological roles of nitrogen‐fixing plants in Europe. As individual lineages may move according to their shifting ecological niche—responding to warmer, drier, wetter or more disturbed conditions—we anticipate substantial range expansions, shifts or contractions. While climate is the dominant driver, these trajectories will also be modulated by non‐climatic factors such as dispersal limitations, habitat fragmentation and soil suitability. Crucially, these range shifts will fundamentally alter ecosystem functions: newly colonised regions will experience novel nitrogen inputs, while areas where nitrogen fixers are lost will face a decline in nutrient cycling capacity. Furthermore, the ongoing expansion of invasive alien nitrogen‐fixing trees and shrubs, such as *Robinia pseudoacacia, Acacia* spp., *Gleditsia triacanthos* and *Amorpha fruticosa*, may increasingly alter soil nutrient regimes and accelerate vegetation succession (Rice *et al*. 2004; Lazzaro *et al*. 2018; Busk & Svenning 2025). These invasive trees reinforce dominance of woody plants at the expense of native open‐habitat specialists. Consequently, the balance between warming‐driven range shifts and biological invasions will be critical in determining the future roles of nitrogen‐fixing lineages in Europe's vegetation and nutrient cycles.

### Conclusion

Our study reveals striking ecological and biogeographical divergence among nitrogen‐fixing plant lineages in Europe. Despite sharing a key functional innovation that overcomes nitrogen limitation, these plants occupy largely different environmental spaces. Non‐IRLC egumes are confined mainly to nutrient‐poor, warm and dry habitats, which are suitable for their rhizobial symbionts and where the N‐fixation provides a strong benefit. These associations with environmental gradients are relaxed or even absent in IRLC legumes, with their advanced control over the rhizobial symbiosis. Actinorhizal plants show pronounced lineage‐specific differentiation, with *Alnus glutinosa* dominating nutrient‐rich, wet environments, while other lineages exhibit distinct geographic and ecological niches. These contrasts demonstrate that a shared, and even phylogenetically conserved, functional trait such as symbiotic nitrogen fixation has given rise to remarkably diverse ecological strategies.

## AUTHOR CONTRIBUTIONS

JT and IA conceived the idea. NF, IA, J‐CS, JPC, TT and JT developed the analyses for the study. IA and NF prepared the data for the study. NF, JPC and JT performed the analyses. J‐CS, IB, JAC, JD, and EG contributed vegetation‐plot data. NF, TT and JT wrote the manuscript. All authors commented on the manuscript drafts and approved the final version.

## Supporting information


**Appendix S1.** Data selection and final dataset included in the study.
**Appendix S3.** Results of the RDA forward selection.
**Appendix S4.** Maps of the representation of symbiotic lineages of legumes and actinorhizal plants in EUNIS habitat types.
**Appendix S5.** Maps for the climatic variables used in the study.
**Appendix S6.** Details on the fitting and evaluation of the boosted regression tree analysis, the partial dependence plots for all analyses and testing for spatial autocorrelation.
**Appendix S7.** Additional information for the EIVE values and the modified permutation test for EIVE values.

**Appendix S2.** Additional information on the most common habitat types for all nitrogen‐fixing plant species in the dataset and the occurrence of nitrogen‐fixing species in EUNIS habitat types.

## Data Availability

The data and code used for the analyses are available at https://doi.org/10.5281/zenodo.19398191. Climate data were obtained from the CHELSA Bioclim (https://www.chelsa‐climate.org/datasets/chelsa_bioclim). Ecological Indicator Values for Europe are available at https://vcs.pensoft.net/article/98324/.
