# Supplementary material for: Ecological niches and biogeography of nitrogen‐fixing plants in Europe
Source: Plant Biol (Stuttg). 2026 May 14;28(5):1349–60. doi: 10.1111/plb.70230 (PMC13358710; doi:10.1111/plb.70230)
Supplement: Supplementary file 1 — Appendix S1. Data selection and final dataset included in the study. Appendix S3. Results of the RDA forward selection. Appendix S4. Maps of the representation of symbiotic lineages of legumes and actinorhizal plants in EUNIS habitat types. Appendix S5. Maps for the climatic variables used in the study. Appendix S6. Details on the fitting and evaluation of the boosted regression tree analysis, the partial dependence plots for all analyses and testing for spatial autocorrelation. Appendix S7. Additional information for the EIVE values and the modified permutation test for EIVE values. [file PLB-28-1349-s002.docx]

**APPENDICES S1; S3– S7**

**Supporting information for**

Fahs, N., Axmanová, I., Svenning, J.–C., Těšitelová, T., Padullés Cubino, J., Biurrun, I., Campos, J.A., Dengler, J., Garbolino, E. and Těšitel, J.: Ecological niches and biogeography of nitrogen-fixing plants in Europe

**Table of contents**

**APPENDIX S1.** Data selection and final dataset included in the study

**APPENDIX S3.** Results of the RDA forward selection

**APPENDIX S4.** Maps of the representation of symbiotic lineages of legumes and actinorhizal plants in EUNIS habitat types

**APPENDIX S5.** Maps for the climatic variables used in the study

**APPENDIX S6.** Details on the fitting and evaluation of the boosted regression tree analysis, the partial dependence plots for all analyses, and testing for spatial autocorrelation

**APPENDIX S7.** Additional information for the EIVE values and the modified permutation test for EIVE values

**APPENDIX S1.** Data selection and final dataset included in the study

**Data selection and filtering**

Official data request criteria for the dataset obtained from the European Vegetation Archive included: (i) georeference of plots (≤10 km uncertainty), (ii) cover-abundance values, and (iii) exclusion of aquatic vegetation. We further excluded plots from Belarus, Georgia, Russia and Turkey. To minimise spatial pseudoreplication, we applied geographical stratification, selecting only one plot from each pair within 1 km with a compositional similarity >0.8 (Bray-Curtis dissimilarity weighted by cover). We excluded all plots recorded before 1970 to reflect the most recent occurrences. To be as comprehensive as possible in geographic and ecological space, we kept all remaining plots, despite variations in plot size or missing information. We accounted for the variation in plot size by using relative cover values. Plot sizes ranged from 1–1000 m², with detailed ranges by habitat type provided in Appendix S2.2. 168,800 plots of the final dataset had no plot size information. The final dataset also included 96,537 plots without information on the sampling date and 110,658 plots without information on georeference uncertainty.

**Table S1.1.** The contribution of individual vegetation databases to the dataset used in this study. For each database, the total number of plots and their proportion in the final selection (N = 707,673) are provided.

| Database | Database custodian | Number of plots | Percentage of dataset (%) |
| --- | --- | --- | --- |
| AMS-VegBank | Alessandro Chiarucci | 8278 | 1.170 |
| Albanian Vegetation Database | Michele De Sanctis | 435 | 0.061 |
| Ammophiletea Database | Corrado Marcenò | 2468 | 0.349 |
| Austria_VINCA | Wolfgang Willner | 33077 | 4.674 |
| Balkan Dry Grassland Database | Kiril Vassilev | 5851 | 0.827 |
| Balkan Vegetation Database | Kiril Vassilev | 8773 | 1.240 |
| Basque Country Database | Idoia Biurrun | 13632 | 1.926 |
| Beech Forest DB SE Balkan | Aleksander Marinšek | 809 | 0.114 |
| Belgium-INBOVEG | Els De Bie | 11482 | 1.623 |
| Belgium-forest | Els De Bie | 3382 | 0.478 |
| Britain_nvcd | John S. Rodwell | 23159 | 3.273 |
| Bulgarian Vegetation Database | Iva Apostolova | 1469 | 0.208 |
| CBNA | Sylvain Abdulhak | 2643 | 0.373 |
| CircumMed Pine Forest database | Gianmaria Bonari | 3172 | 0.448 |
| Croatia_mix | Zvjezdana Stančić | 2515 | 0.355 |
| Croatian Vegetation Database | Željko Škvorc | 8632 | 1.220 |
| Czechia_nvd | Milan Chytrý | 60733 | 8.582 |
| Eastern European Steppe Database | Denys Vynokurov | 3256 | 0.460 |
| European Boreal Forest Database 1 | Anni Kanerva Jašková | 39 | 0.006 |
| European Boreal Forest Database 2 | Anni Kanerva Jašková | 5420 | 0.766 |
| European Coastal Vegetation Database-A | John Janssen | 1502 | 0.212 |
| European Mire VDB | Tomáš Peterka | 5406 | 0.764 |
| European Weed Vegetation Database | Filip Küzmič | 898 | 0.127 |
| France_SOPHY | Emmanuel Garbolino | 88539 | 12.511 |
| Germany Coastal VDB | Maike Isermann | 8 | 0.001 |
| Germany Vegetweb 1 | Florian Jansen | 5057 | 0.715 |
| Germany Vegetweb 2 | Friedemann Goral | 361 | 0.051 |
| Germany Vegetweb 3 | Friedemann Goral | 2 | <0.001 |
| Germany_gvrd | Ute Jandt | 27933 | 3.947 |
| Germany_vegmv | Florian Jansen | 20790 | 2.938 |
| GrassVeg.DE | Ricarda Pätsch | 6253 | 0.884 |
| Gravel Bar Database_Europe | Veronika Kalníková | 718 | 0.101 |
| Greece_forests | Ioannis Tsiripidis | 623 | 0.088 |
| Greece_nat | Panayotis Dimopoulos | 4083 | 0.577 |
| High Mediterranean Mountains Database | Gianpietro Giusso del Galdo | 553 | 0.078 |
| Hungary | János Csiky | 2216 | 0.313 |
| Ireland_nvd | Úna FitzPatrick | 17746 | 2.508 |
| Italy_HabItAlp | Laura Casella | 3486 | 0.493 |
| Italy_UniRoma | Emiliano Agrillo | 21425 | 3.028 |
| Italy_mires | Laura Casella | 566 | 0.080 |
| Kriti | Erwin Bergmeier | 2550 | 0.360 |
| Latvian Grassland VDB | Solvita Rūsiņa | 4484 | 0.634 |
| Lithuania | Valerius Rašomavičius | 4639 | 0.656 |
| Macedonia | Renata Ćušterevska | 162 | 0.023 |
| Masaryk University Database 1 | Milan Chytrý | 326 | 0.046 |
| Netherlands | Stephan Hennekens | 76299 | 10.782 |
| Netherlands Military sites | Iris de Ronde | 8092 | 1.143 |
| Nordic Vegetation Database 1 | Jonathan Lenoir | 3325 | 0.470 |
| Nordic Vegetation Database 2 | Jonathan Lenoir | 510 | 0.072 |
| Nordic_Baltic EDGG | Jürgen Dengler | 1807 | 0.255 |
| Poland | Zygmunt Kącki | 41452 | 5.858 |
| Poland Forest Database | Remigiusz Pielech | 3173 | 0.448 |
| RanVegDunes | Alicia Acosta | 1055 | 0.149 |
| Romania Grassland Database | Eszter Ruprecht | 12363 | 1.747 |
| Romania_indreica | Adrian Indreica | 5920 | 0.837 |
| SE Europe Forest DB | Andraž Čarni | 2054 | 0.290 |
| Schleswig-Holstein Db | Joachim Schrautzer | 2023 | 0.286 |
| Scottish Coastal Survey | Robin Pakeman | 5231 | 0.739 |
| Scottish Vegetation Survey | Ruth Mitchell | 4754 | 0.672 |
| Serbia_grasslands | Svetlana Aćić | 3190 | 0.451 |
| Serbian VDB | Mirjana Krstivojević Ćuk | 477 | 0.067 |
| Serra da Estrella database | Jan Jansen | 821 | 0.116 |
| Slovakia_nvd | Milan Valachovič | 21776 | 3.077 |
| Slovenia | Urban Šilc | 12311 | 1.740 |
| Spain_sivim | Xavier Font | 1573 | 0.222 |
| Spain_sivim_Catalonia | Xavier Font | 2244 | 0.317 |
| Spain_sivim_alpine | Borja Jiménez-Alfaro | 4227 | 0.597 |
| Spain_sivim_floodplains | Idoia Biurrun | 3253 | 0.460 |
| Spain_sivim_forests | Juan Antonio Campos | 3586 | 0.507 |
| Spain_sivim_grasslands | Maria Pilar Rodríguez-Rojo | 8386 | 1.185 |
| Spain_sivim_sclerophyllous | Federico Fernández-González | 2631 | 0.372 |
| Spain_sivim_sclerophyllous_pinus | Federico Fernández-González | 5615 | 0.793 |
| Spain_sivim_scrubs | Rosario G Gavilán | 2403 | 0.340 |
| Spain_sivim_wetlands | Aaron Pérez-Haase | 3561 | 0.503 |
| Switzerland Grassland DB | Ariel Bergamini | 7790 | 1.101 |
| Switzerland_forests | Thomas Wohlgemuth | 7442 | 1.052 |
| Turkey Forest Database | Ali Kavgacı | 44 | 0.006 |
| UK Floodplain Meadows Database | Irina Tatarenko | 10950 | 1.547 |
| Ukraine Grassland Database A | Anna Kuzemko | 5768 | 0.815 |
| Ukraine Halophytic Coastal VDB | Tetiana Dziuba | 495 | 0.070 |
| Ukraine Psammophytic VDB | Tetiana Dziuba | 610 | 0.086 |
| Ukraine_onyshchenko | Viktor Onyshchenko | 1736 | 0.245 |
| VegFrance | Jan-Bernard Bouzillé | 2529 | 0.357 |
| VegItaly | Roberto Venanzoni | 5690 | 0.804 |
| Viola db | Angela Stanisci | 950 | 0.134 |
| WetVegEurope Database | Flavia Landucci | 6 | 0.001 |

Further information on the databases can be found at <https://euroveg.org/eva-database/databases>.

**Table S1.2.** Contribution of vegetation plots to the final study dataset by country. Numbers indicate the total plots retained after selection (N = 707,673).

| Country | Plot number in the final dataset | Percentage of the final dataset (%) |
| --- | --- | --- |
| Albania | 452 | 0.064 |
| Andorra | 413 | 0.058 |
| Austria | 32165 | 4.545 |
| Belgium | 17607 | 2.488 |
| Bosnia-Herzegovina | 2055 | 0.290 |
| Bulgaria | 13395 | 1.893 |
| Croatia | 11349 | 1.604 |
| Czech Republic | 59962 | 8.473 |
| Denmark | 10 | 0.001 |
| Estonia | 2210 | 0.312 |
| Faroe Islands | 16 | 0.002 |
| Finland | 2494 | 0.352 |
| France | 90026 | 12.721 |
| Germany | 63412 | 8.961 |
| Greece | 9551 | 1.350 |
| Hungary | 2375 | 0.336 |
| Iceland | 380 | 0.054 |
| Ireland | 17867 | 2.525 |
| Italy | 45696 | 6.457 |
| Kosovo | 442 | 0.062 |
| Latvia | 8524 | 1.205 |
| Lithuania | 4730 | 0.668 |
| Luxembourg | 157 | 0.022 |
| Macedonia | 793 | 0.112 |
| Malta | 419 | 0.059 |
| Moldova | 182 | 0.026 |
| Monaco | 18 | 0.003 |
| Montenegro | 142 | 0.020 |
| Netherlands | 83960 | 11.864 |
| Norway | 3276 | 0.463 |
| Poland | 45900 | 6.486 |
| Portugal | 2566 | 0.363 |
| Romania | 18704 | 2.643 |
| San Marino | 61 | 0.009 |
| Serbia | 4796 | 0.678 |
| Slovak Republic | 22142 | 3.129 |
| Slovenia | 13793 | 1.949 |
| Spain | 46946 | 6.634 |
| Svalbard and Jan Mayen Is | 162 | 0.023 |
| Sweden | 2231 | 0.315 |
| Switzerland | 20037 | 2.831 |
| Ukraine | 11837 | 1.673 |
| United Kingdom | 44420 | 6.277 |

Figures S1.1–S1.7 present the distribution of plot numbers in the dataset across regions for each broad EUNIS habitat type considered in the analyses.

**Fig. S1.1.** Total number of vegetation plots in the final dataset for EUNIS habitat type M (Coastal Saltmarshes) per grid cell. Grid definition as described in the Methods of the manuscript.

**Fig. S1.2.** Total number of vegetation plots in the final dataset for EUNIS habitat type N (Coastal Sand and Cliff Habitats) per grid cell. Grid definition as described in the Methods of the manuscript .

**Fig. S1.3.** Total number of vegetation plots in the final dataset for EUNIS habitat type Q (Wetlands) per grid cell. Grid definition as described in the Methods of the manuscript.

**Fig. S1.4.** Total number of vegetation plots in the final dataset for EUNIS habitat type R (Grasslands) per grid cell. Grid definition as described in the Methods of the manuscript.

**Fig. S1.5.** Total number of vegetation plots in the final dataset for EUNIS habitat type S (Heathlands, Scrub and Tundra) per grid cell. Grid definition as described in the Methods of the manuscript.

**Fig. S1.6.** Total number of vegetation plots in the final dataset for EUNIS habitat type T (Forests) per grid cell. Grid definition as described in the Methods of the manuscript.

**Fig. S1.7.** Total number of vegetation plots in the final dataset for EUNIS habitat type U (Inland Sparsely Vegetated Habitats) per grid cell. Grid definition as described in the Methods of the manuscript.

**APPENDIX S3.** Results of the RDA forward selection

We conducted a forward selection of climatic variables (bio1–bio19) using redundancy analysis (RDA) to identify predictors for subsequent analyses. Variables were log-transformed as described in Table S2.4. Climatic values were used as response variables. The forward selection was carried out with the *vegan* package in R (version 4.3.0; R Core Team, 2023), applying centering and scaling to unit variance within the rda function. Based on AIC criteria, the following variables were retained: mean temperature of the warmest quarter (bio10), temperature seasonality (bio4), annual precipitation (bio12), precipitation seasonality (bio15), mean diurnal temperature range (bio2), and mean precipitation of the warmest quarter (bio18). When two or more variables showed comparable AIC values, we prioritised those reflecting conditions during the main growing season of most plant species. The constrained axes explained 95.3% of the total variability (adjusted R²).

**Fig. S3.1.** Pairwise relationships among the climatic variables included in the analysis. The figure displays scatterplots with LOESS trend lines in the lower triangle, density plots along the diagonal (LOESS smoothed), and Pearson correlation coefficients (ranging from -1 to 1) in the upper triangle. See Table S2.4 for details on the selected climatic variables.

References:

R Core Team. (2023). R: A language and environment for statistical computing*.* R Foundation for Statistical Computing, Vienna, Austria. *Open Journal of Statistics*, *13*(02).

**APPENDIX S4.** Maps of the representation of symbiotic lineages of legumes and actinorhizal plants in EUNIS habitat types

Fig. S4.1 shows the relative share of legumes and actinorhizal plants in the species pool per grid cell. Fig. S4.2 to S4.13 show the mean relative cover of symbiotic lineages of nitrogen-fixing plants in Europe according to broad EUNIS habitat types.

(a) (b)

**Fig. S4.1.** Relative share of (a) legumes, and (b) actinorhizal plants in the species pool per grid cell across all EUNIS habitat types. Data is presented per 30 km grid cell (Lambert Azimuthal Equal Area projection, EPSG:3035). Grid definition as described in the Methods of the manuscript.

**Fig. S4.2.** Mean relative cover of symbiotic lineages of legumes and actinorhizal plants per grid cell for the EUNIS habitat type M Coastal Saltmarshes. Data is presented as mean values per 30 km grid cell (Lambert Azimuthal Equal Area projection, EPSG:3035). Grid definition as described in the Methods of the manuscript. Only grid cells with ≥3 vegetation records were included in the analysis. Myricaceae and Coriariaceae are missing due to too few data.

**Fig. S4.3.** Mean relative cover of symbiotic lineages of legumes and actinorhizal plants per grid cell for the EUNIS habitat type N Coastal Sand and Cliff habitats. Data is presented as mean values per 30 km grid cell (Lambert Azimuthal Equal Area projection, EPSG:3035). Grid definition as described in the Methods of the manuscript. Only grid cells with ≥3 vegetation records were included in the analysis. Coriariaceae are missing due to too few data.

**Fig. S4.4.** Mean relative cover of symbiotic lineages of legumes and actinorhizal plants per grid cell for the EUNIS habitat type Q Wetlands. Data is presented as mean values per 30 km grid cell (Lambert Azimuthal Equal Area projection, EPSG:3035). Grid definition as described in the Methods of the manuscript. Only grid cells with ≥3 vegetation records were included in the analysis. Coriariaceae are missing due to too few data.

**Fig. S4.5.** Mean relative cover of symbiotic lineages of legumes and actinorhizal plants per grid cell for the EUNIS habitat type R Grasslands. Data is presented as mean values per 30 km grid cell (Lambert Azimuthal Equal Area projection, EPSG:3035). Grid definition as described in the Methods of the manuscript. Only grid cells with ≥3 vegetation records were included in the analysis.

**Fig. S4.6.** Mean relative cover of symbiotic lineages of legumes and actinorhizal plants per grid cell for the EUNIS habitat type S Heathlands, Scrub and Tundra. Data is presented as mean values per 30 km grid cell (Lambert Azimuthal Equal Area projection, EPSG:3035). Grid definition as described in the Methods of the manuscript. Only grid cells with ≥3 vegetation records were included in the analysis.

**Fig. S4.7.** Mean relative cover of symbiotic lineages of legumes and actinorhizal plants per grid cell for the EUNIS habitat type T Forests. Data is presented as mean values per 30 km grid cell (Lambert Azimuthal Equal Area projection, EPSG:3035). Grid definition as described in the Methods of the manuscript. Only grid cells with ≥3 vegetation records were included in the analysis.

**Fig. S4.8.** Mean relative cover of symbiotic lineages of legumes and actinorhizal plants per grid cell for the EUNIS habitat type U Inland Sparsely Vegetated habitat types. Data is presented as mean values per 30 km grid cell (Lambert Azimuthal Equal Area projection, EPSG:3035). Only grid cells with ≥3 vegetation records were included in the analysis. Grid definition as described in the Methods of the manuscript. Myricaceae and Coriariaceae are missing due to too few data.

**APPENDIX S5.** Maps for the climatic variables used in the study

Similar to the maps illustrating the distribution of symbiotic lineages, we also created maps summarising the spatial patterns of the climatic predictors used in this study. Using the same grid as for the other maps, we calculated the mean value of each climatic variable across all vegetation plots within a grid cell. Unlike the previous approach, cells containing fewer than three plots were retained. For further information, see the Methods section of the manuscript.

**Fig. S5.1.** Mean values of the mean diurnal air temperature range (°C) per grid cell.

**Fig. S5.2.** Mean values of the temperature seasonality (°C/100) per grid cell.

**Fig. S5.3.** Mean values of the mean daily mean air temperatures of the warmest quarter (°C) per grid cell.

**Fig. S5.4.** Mean values of the annual precipitation (mm/a) per grid cell.

**Fig. S5.5.** Mean values of the precipitation seasonality (mm) per grid cell.

**Fig. S5.6.** Mean values of the mean monthly precipitation amount of the warmest quarter (mm/month) per grid cell.

**APPENDIX S6.** Details on the fitting and evaluation of the boosted regression tree analysis, the partial dependence plots for all analyses, and testing for spatial autocorrelation

In Fig. S6.1-S6.3 we present all the partial dependence plots from the boosted regression tree models presented in the main part of the manuscript. Fig. S6.4 shows the results of the boosted regression tree model including the atmospheric nitrogen values. The results were obtained as described in the Methods section of the manuscript.

**Boosted Regression Trees**

Boosted regression tree (BRT) models were fitted following the framework of Elith et al. (2008) using the gbm.step function in the *dismo* package. For each tree-fitting step, 50% of the available data were randomly selected to train the model, while the remaining 50% were used to evaluate predictive performance, thereby improving model robustness through internal validation. A low learning rate (shrinkage) of 0.001 was chosen to ensure that each successive tree made only a small contribution to the overall model, promoting gradual optimisation and reducing the risk of overfitting. The tree complexity parameter was set to 5, allowing the model to capture interactions among predictors up to the fifth order while maintaining reasonable computational demands.

Model performance was assessed using cross-validation statistics generated by the gbm.step function in the dismo package following the approach of Elith et al. (2008). The optimal number of trees was determined automatically by gbm.step using a 10-fold cross-validation procedure. At each iteration, model performance was evaluated based on the mean change in predictive deviance across folds, and the optimal number of trees was identified as the iteration at which this hold-out deviance reached its minimum. Predictive deviance represents the negative log-likelihood of the model predictions on hold-out data; lower values indicate better fit. The mean cross-validated deviance (CV Deviance mean) and its standard error (CV Deviance SE) were calculated across k = 10 folds. The cross-validated correlation (CV Correlation) is the mean Pearson correlation between predicted and observed responses across folds, providing a measure of predictive accuracy for continuous models. For binomial models, the cross-validated Area Under the Curve (CV AUC) quantifies the model’s ability to discriminate between presence and absence. Values near 0.5 indicate random discrimination performance, and values near 1 indicate perfect discrimination. The pseudo-R² (proportion of deviance explained) was calculated as pseudo-R² = 1−(CV mean deviance/null deviance), where the null deviance corresponds to the deviance of a model with only an intercept term. This value approximates the percentage of total variation explained by the BRT model, analogous to an R² statistic. The training deviance and training correlation describe the model fit on the training data subset. These are included for reference but may overestimate predictive performance relative to cross-validation metrics.

Model evaluation statistics reported in Table S6.1 were extracted from the gbm objects generated by gbm.step as described above. These statistics were obtained using the ggPerformance function from the ggBRT package (Jouffray et al., 2019). All reported metrics are fold-based, meaning they are calculated at the cross-validated optimal number of trees determined by the gbm.step procedure.

**Spatial autocorrelation**

To assess potential spatial autocorrelation in model residuals, we used Moran’s I. For all symbiotic lineages 100 plots were randomly selected, and for each, spatial correlograms were computed using all plots located within 2° (~225 km) of the target plot. The resulting correlograms were summarised using LOESS smoothing to visualise the spatial structure of residuals.

**Fig. S6.1.** Partial dependence plots from the boosted regression tree models (BRT) presented in the main part of the manuscript showing the effects of climatic variables (a-f) on the abundance weighted occurrence probability of legumes in Europe. Lines represent fitted response curves derived from the models. Precipitation-related variables (d-f) are displayed on log-scaled axes. Variable importance values for the models are provided in the Results section of the main text.

**Fig. S6.2.** Partial dependence plots from the boosted regression tree models (BRT) showing the effects of climatic variables (a-f) on the abundance weighted occurrence probability of *Alnus* in Europe. Lines represent fitted response curves derived from the models. Precipitation-related variables (d-f) are displayed on log-scaled axes. Variable importance values for the models are provided in the Results section of the main text.

**Fig. S6.3.** Partial dependence plots from the boosted regression tree models (BRT) showing the effects of climatic variables (a-f) on the abundance weighted occurrence probability of nitrogen-fixing Myricaceae, Elaeagnaceae, and Coriariaceae in Europe. Lines represent fitted response curves derived from the models. Precipitation-related variables (d-f) are displayed on log-scaled axes. Variable importance values for the models are provided in the Results section of the main text.

**Table S6.1.** Evaluation statistics of the boosted regression tree models for symbiotic lineages of legumes and actinorhizal plants of the BRTs presented in the main part of the manuscript.

| **Model** | **Optimal** | **CV Deviance** | | **CV Correlation** | | **CV AUC** | |  | **Training dataset** | |
| --- | --- | --- | --- | --- | --- | --- | --- | --- | --- | --- |
|  | **no. of trees** | **Mean** | **SE** | **Mean** | **SE** | **AUC** | **SE** | **Pseudo R^2^** | **Deviance** | **Correlation** |
| IRLC legumes | 7450 | 1.282 | <0.001 | 0.263 | <0.001 | 0.652 | <0.001 | 0.050 | 1.281 | 0.265 |
| non-IRLC legumes | 7300 | 1.086 | <0.001 | 0.326 | <0.001 | 0.708 | 0.001 | 0.091 | 1.085 | 0.327 |
| Alnus (exc. *A. glutinosa*) | 16600 | 0.108 | <0.001 | 0.283 | 0.003 | 0.884 | 0.001 | 0.227 | 0.107 | 0.301 |
| *Alnus glutinosa* | 16500 | 0.274 | <0.001 | 0.256 | 0.002 | 0.788 | 0.001 | 0.133 | 0.272 | 0.265 |
| Myricaceae | 23700 | 0.026 | <0.001 | 0.387 | 0.008 | 0.963 | 0.002 | 0.412 | 0.025 | 0.434 |
| Elaeagnaceae | 18000 | 0.031 | <0.001 | 0.460 | 0.005 | 0.970 | 0.002 | 0.497 | 0.030 | 0.481 |
| Coriariaceae | 19250 | 0.009 | <0.001 | 0.469 | 0.013 | 0.989 | 0.001 | 0.550 | 0.008 | 0.538 |

**Fig. S6.4.** Spatial correlograms of model residuals for each symbiotic lineage of legumes and actinorhizal plants, derived from boosted regression tree (BRT) models presented in the main part of the manuscript. Moran’s I statistic was calculated for plots within a 2° radius (~225 km) of 100 randomly selected plots, distributed across the sampled regions, based on a random subset representing 10% of all plots. Red lines show overall mean trends across repetitions, fitted using LOESS regression.

**Fig. S6.5.** Partial dependence plots from the boosted regression tree models (BRT) including atmospheric nitrogen (not included in the main part of the study) showing the effects of climatic variables (a-f) and atmospheric nitrogen (g-h) on the abundance weighted occurrence probability of legumes and actinorhizal plants in Europe. Lines represent fitted response curves derived from the models. Precipitation-related variables and atmospheric nitrogen values (d-f) are displayed on log-scaled axes. Variable importance values for the models are provided in Table S6.1.

**Table S6.2.** Relative influence (%) of environmental predictors on the abundance weighted occurrence probability of nitrogen-fixing symbiotic lineages in Europe derived from BRTs including atmospheric nitrogen and climate (not included in the main part of the study).

|  | Legumes | Actinorhizal plants |
| --- | --- | --- |
| Mean diurnal air temperature range (°C) | 21.6 | 16.1 |
| Temperature seasonality (°C/100) | 11.5 | 18.7 |
| Mean daily mean air temperatures of the warmest quarter (°C) | 29.5 | 16.5 |
| Annual precipitation amount (log10(mm)) | 5.6 | 11.8 |
| Precipitation seasonality (log10(mm)) | 7.2 | 7.9 |
| Mean monthly precipitation amount of the warmest quarter (log10(mm)) | 19.0 | 13.7 |
| Mean atmospheric nitrogen (µg/m³) | 3.3 | 9.6 |
| Total atmospheric nitrogen (µg/m³) | 2.2 | 5.7 |

The score values of each model are scaled, summing up to 100% (numbers displayed are rounded).

References

Elith, J., Leathwick, J. R., & Hastie, T. (2008). (2008). A working guide to boosted regression trees. *Journal of Animal Ecology, 77*(4), 802–813. [https://doi.org/10.1111/j.1365-2656.2008.01390.x](https://doi.org/10.1111/j.1365-2656.2008.01390.x)

Jouffray, J.-B., Wedding, L. M., Norström, A. V., Donovan, M. K., Williams, G. J., Crowder, L. B., … Nyström, M. (2019). Parsing human and biophysical drivers of coral reef regimes. *Proceedings of the Royal Society B: Biological Sciences*, 286(1896), 20182544. <https://doi.org/10.1098/rspb.2018.2544>

**APPENDIX S7.** Additional information for the EIVE values and the modified permutation test for EIVE values

Fig. S7.1-S7.9 show the results of the modified permutation test (Zeleny & Schaffers, 2012) comparing Environmental Indicator Values for Europe (EIVE) between plots with and without symbiotic lineages of legumes and actinorhizal plants. The red dashed line indicates the observed difference, while the grey histogram shows the distribution of differences obtained from 999 random permutations of species EIVE values. For more information on the methods, see Methods of the manuscript.

**Tab. S7.1.** Mean Environmental Indicator Values for Europe (EIVE) across plots containing symbiotic lineages.

|  | EIVE soil nitrogen | EIVE soil moisture | EIVE soil reaction | EIVE temperature | EIVE light |
| --- | --- | --- | --- | --- | --- |
| IRLC legumes | 4.52 | 4.41 | 6.07 | 4.24 | 6.91 |
| non-IRLC legumes | 3.93 | 4.08 | 6.07 | 4.40 | 7.05 |
| *Alnus* (exc. *A. glutinosa*) | 5.32 | 5.08 | 5.88 | 3.81 | 5.36 |
| *Alnus glutinosa* | 5.53 | 5.63 | 5.80 | 4.12 | 5.65 |
| Myricaceae | 2.99 | 6.38 | 4.14 | 3.85 | 7.14 |
| Elaeagnaceae | 4.90 | 4.32 | 6.09 | 4.38 | 7.05 |
| Coriariaceae | 3.82 | 3.47 | 6.76 | 5.52 | 6.90 |

For each calculation, EIVE values of the plants of the focal lineage were excluded. Further details on the data source and calculation methods are provided in the Methods section of the manucript.

**Fig. S7.1.** Distribution of the differences between plots with and without legumes, generated by calculating EIVE plot values after permutation of species values. We used 999 permutations. The red dashed line shows the actual difference between plots with and without legumes.

**Fig. S7.2.** Distribution of the differences between plots with and without actinorhizal plants, generated by calculating EIVE plot values after permutation of species values. We used 999 permutations. The red dashed line shows the actual difference between plots with and without actinorhizal plants.

**Fig. S7.3.** Distribution of the differences between plots with and without IRLC legumes, generated by calculating EIVE plot values after permutation of species values. We used 999 permutations. The red dashed line shows the actual difference between plots with and without IRLC legumes.

**Fig. S7.4.** Distribution of the differences between plots with and without non-IRLC legumes, generated by calculating EIVE plot values after permutation of species values. We used 999 permutations. The red dashed line shows the actual difference between plots with and without non-IRLC legumes.

**Fig. S7.5.** Distribution of the differences between plots with and without *Alnus* (exc. *Alnus glutinosa*), generated by calculating EIVE plot values after permutation of species values. We used 999 permutations. The red dashed line shows the actual difference between plots with and without *Alnus* (exc. *Alnus glutinosa*).

**Fig. S7.6.** Distribution of the differences between plots with and without *Alnus glutinosa*, generated by calculating EIVE plot values after permutation of species values. We used 999 permutations. The red dashed line shows the actual difference between plots with and without *Alnus glutinosa*.

**Fig. S7.7.** Distribution of the differences between plots with and without Myricaceae, generated by calculating EIVE plot values after permutation of species values. We used 999 permutations. The red dashed line shows the actual difference between plots with and without Myricaceae.

**Fig. S7.8.** Distribution of the differences between plots with and without Elaeagnaceae, generated by calculating EIVE plot values after permutation of species values. We used 999 permutations. The red dashed line shows the actual difference between plots with and without Elaeagnaceae.

**Fig. S7.9.** Distribution of the differences between plots with and without Coriariaceae, generated by calculating EIVE plot values after permutation of species values. We used 999 permutations. The red dashed line shows the actual difference between plots with and without Coriariaceae.
